# Supplementary material for: Beyond the hype: deep neural networks outperform established methods using a ChEMBL bioactivity benchmark set
Source: J Cheminform. 2017 Aug 14;9:45. doi: 10.1186/s13321-017-0232-0 (PMC5555960; doi:10.1186/s13321-017-0232-0)
Supplement: Supplementary file 1 — Additional file 1:. Contains information supporting the work published in this paper in the form of 8 figures, 8 tables, and a methods section for the work performed in Pipeline Pilot. [file 13321_2017_232_MOESM1_ESM.docx]

**Beyond the Hype: Deep Neural Networks Outperform Established Methods Using A ChEMBL Bioactivity Benchmark Set**

Eelke B. Lenselink^1†^, Niels ten Dijke^2^, Brandon Bongers^1^, George Papadatos^3^, Herman W.T. van Vlijmen^1^, Wojtek Kowalczyk^2^, Adriaan P. IJzerman^1^, and Gerard J.P. van Westen^1,* †^

^1^ Division of Medicinal Chemistry, Leiden Academic Centre for Drug Research, Leiden University, P.O. Box 9502, 2300 RA Leiden, The Netherlands

^2^ Leiden Institute of Advanced Computer Science, Leiden University, P.O. Box 9512, 2300 RA Leiden, The Netherlands

^3^ European Molecular Biology Laboratory, European Bioinformatics Institute (EMBL-EBI), Wellcome Genome Campus, Hinxton, Cambridge, United Kingdom

Author email addresses: EB Lenselink [e.b.lenselink@lacdr.leidenuniv.nl](mailto:e.b.lenselink@lacdr.leidenuniv.nl) , N ten Dijke [nielstendijke@gmail.com](mailto:nielstendijke@gmail.com) , B Bongers [b.j.bongers@lacdr.leidenuniv.nl](mailto:b.j.bongers@lacdr.leidenuniv.nl) , G Papadatos [george.x.papadatos@gsk.com](mailto:george.x.papadatos@gsk.com) , HWT van Vlijmen [hvvlijme@its.jnj.com](mailto:hvvlijme@its.jnj.com) , W Kowalczyk [w.j.kowalczyk@liacs.leidenuniv.nl](mailto:w.j.kowalczyk@liacs.leidenuniv.nl) , AP IJzerman [ijzerman@lacdr.leidenuniv.nl](mailto:ijzerman@lacdr.leidenuniv.nl) , GJP van Westen [gerard@gjpvanwesten.nl](mailto:gerard@gjpvanwesten.nl)

Methods page 3,4

Figure 1: Growth in ChEMBL data page 5

Figure 2: Distribution of High quality data in ChEMBL page 6

Figure 3: Distribution of actives/inactives uSup.ng different cutoffs page 7

Figure 4: Plot of performance of the QSAR versus PCM DNN page 8

Figure 5: Training time in the in hours plotted against the performance page 9

Figure 6: Overview of DNN finetuning page 10

Figure 7: Distribution L1 and L2 targets page 11

Figure 8: Train/validation loss plot of a DNN page 12

Table 1: Results for the random split validation page 13

Table 2: Results for the temporal split validation page 14

Table 3: Results for the training time page 15

Table 4: overview of p values obtained with Students T test test. page 16

Table 5: overview of p values obtained with Fisher F test. page 17

Table 6: overview of p values obtained with Wilcoxon signed rank test. page 18

Table 7: overview of p values obtained with Kolgomorov – Smirnov test. page 19

Table 8: Impact of including inactive data points for the NB page 20

References page 21

**Methods**

Algorithms used in Pipeline Pilot as shown in the SI are described in this section below.

Chemical descriptors. Extended Circular FingerPrints with the same radius and length (ECFP_6) were chosen [[1](#_ENREF_1), [2](#_ENREF_2)]. According to Landrum the RDKit implementation of circular fingerprints is very similar but not identical as was demonstrated in the RDKit user group meeting [[3](#_ENREF_3)].

The NB categorical model trained in PP was constructed in the following way; Continuous variable descriptors were divided in 10 equipopulated bins. Both the active and inactive data points were used by relabeling targets to active and inactive target classes (e.g. P29274_active and P29274_inactive for the adenosine A_2A_ receptor). For every target class NB scores were transformed into z-scores (compared to the mean bayesian score of actives for a given target and the mean bayesian score of inactives for a given target) [[4](#_ENREF_4), [5](#_ENREF_5)]. Subsequently z-scores of the inactive class were subtracted from the active class yielding one z-score for every target. Hence, data points with z-scores higher or equal to 0 were assigned to the active class of a target and lower than 0 to the inactive class.

RF trained in PP used R package randomForest, as has been done before [[6](#_ENREF_6)]. The following settings were used: 1000 trees, 30% of the features were randomly selected to choose the best splitting attribute from, with no limit on the maximum depth of the tree. For scikit-learn the same settings were used except for the multi-class models where the depth of the tree was set to 10 due to memory limitations (>120 GB memory usage). For the multiclass RF, a probability of each class (both active/inactive) was calculated for each entry. The highest probability was chosen as the predicted label and compared to actual experimental label (e.g. for adenosine: A_2A__active, A_2B__inactive).

SVM were trained in PP / R used R package e1071, as was done before.[[7](#_ENREF_7)] The following settings were used: radial basis function kernel (exp(-Gamma * |x' - x|^2)), Gamma was set at 1 / number of descriptors. Cost was set at 1, and epsilon was set at 0.1. For scikit-learn the SVC class of the scikit’s svm package with the same settings was used. For LR trained in PP / R standard R was used and method was set to generalized linear model (glm). IncludeInteractionTerms was set to False and the MaxInteractionOrder was set at 2. For binary class implementations simple ‘active’ and ‘inactive’ classes were used as described above. The multiclass implementation consisted of a concatenation of the binary class and protein modeled as described by Uniprot accession (e.g. ‘active_P29274’ and ‘inactive_P29274’).

***Figure S1****: Growth of data points in ChEMBL. The number of datapoints (in millions) was used; every subsequent point represents a novel ChEMBL release.*


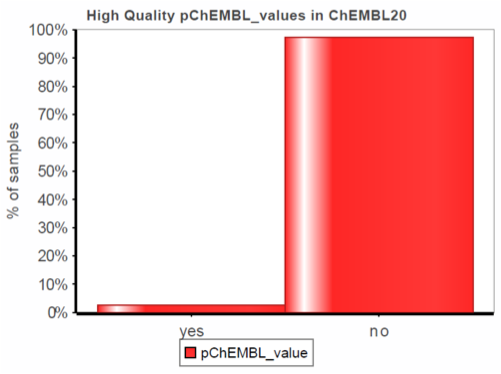


***Figure S2:*** *Total data is reduced to 341,517 data points (or approximately 2.5 %) if we limit our selection to the high quality data in ChEMBL (pChembl values) and calculate the median value for duplicates.*


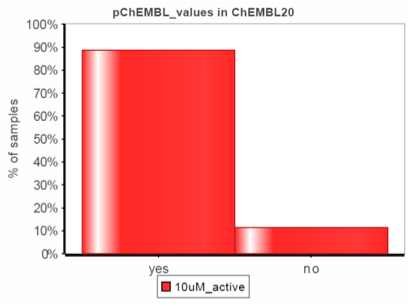


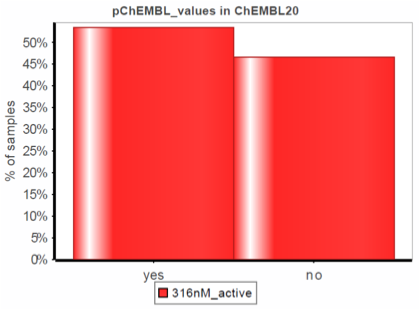


***Figure S3****: Selecting an activity threshold better than pChEMBL 5 (10 μM) (top plot), almost 90% of the data points represents the ‘active’ class. Conversely, using a threshold of 6.5 (corresponding to 300 nM), the division is rougly equal (bottom plot).*


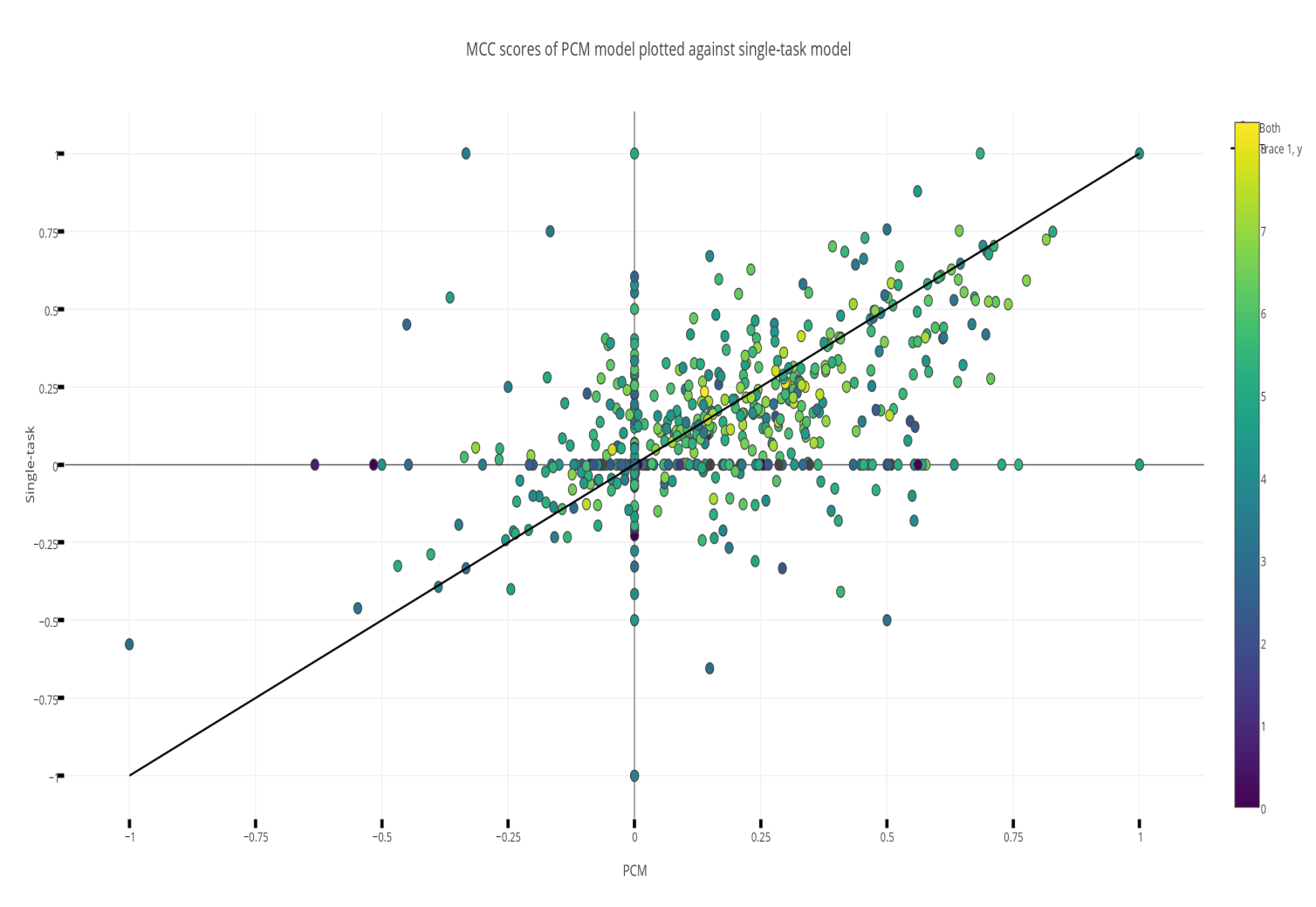


***Figure S4 :*** *Improved performance of PCM over Single Task QSAR. The plot shows the Matthews Correlation Coefficient of the targets of the PCM model on the x-axis against the single-task neural network on the y-axis, where each point is the score for one target. The color represents the natural logarithm of the number of instances in the training set for that particular target. As can be seen, many of the targets with few data points in the training set (blue color) show up on the positive side of the x-axis, which means that PCM outperforms the single-task model in these cases. Hence PCM seems to be able to extrapolate to targets close in target space, when little data is available. However it should be noted that based on this plot it can also be concluded that PCM is not able to solve all these situations as blue dots on the y-axis (PCM MCC = 0) are also seen, and dots on the negative side are also observed. A potentially interesting follow up would be a further exploration of protein descriptors in order to accurately capture the similarity between these targets.*


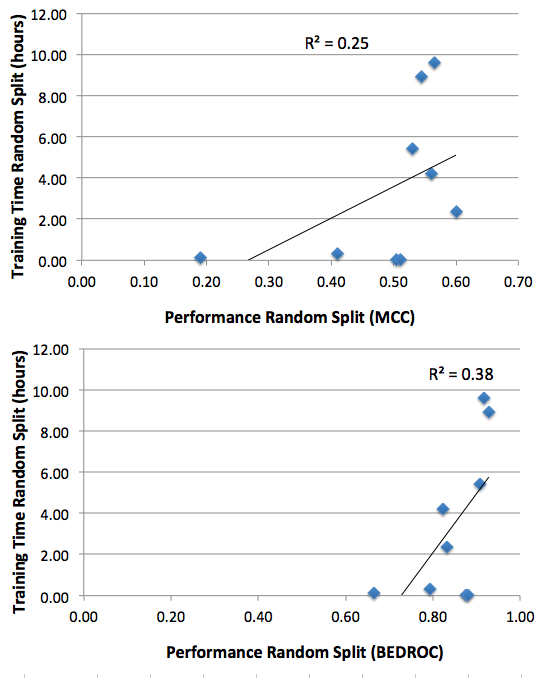


A

B

***Figure S5:*** *Training time in the random split in hours plotted against the performance as MCC (A) and BEDROC (B). Though a weak trend can be observed in both plots (R^2^ 0.25 and 0.38 respectively), there appears to be no direct correlation.*
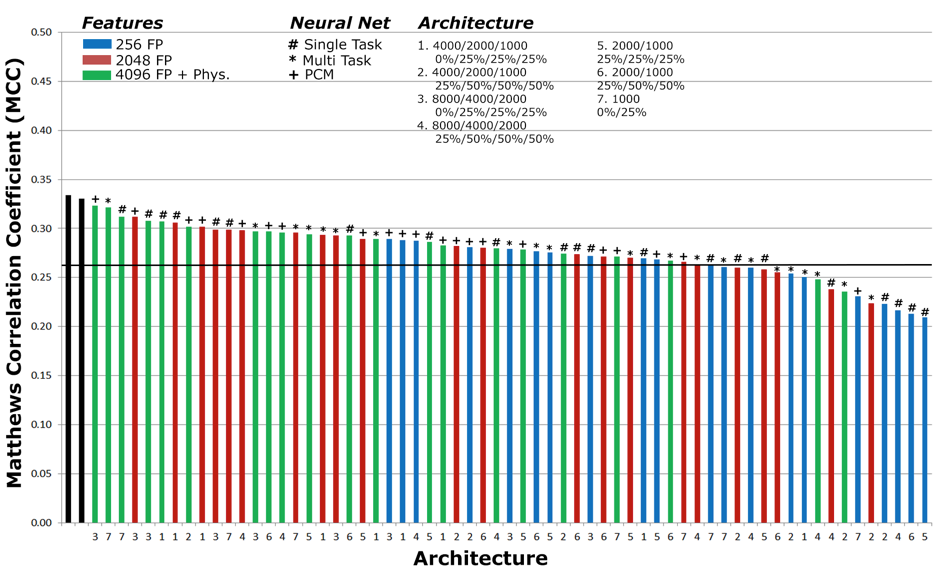


***Figure S6****: DNN tuning, performance of the 63 different networks. DNN are colored by the number of features, the symbol indicates the type of DNN (PCM/single-task/multi-task). The architecture is shown beneath the graphs. The black bars represent average and majority vote ensembles (left and right respectively) The black line represent the previous best performance.*


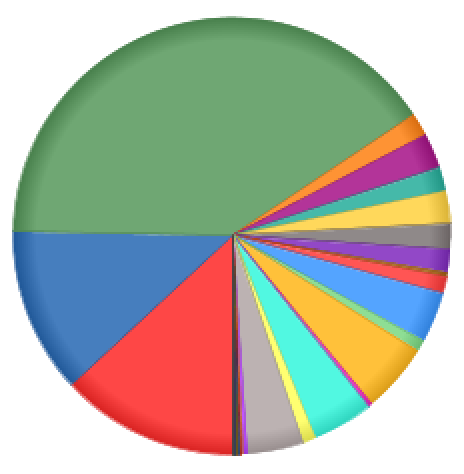

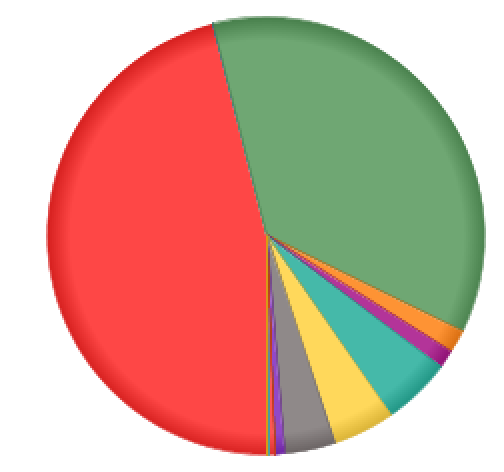


B

A

***Figure S7: Distribution of the L1 (A) and L2 (B) targets from ChEMBL visualized.*** *(A) most dominant are enzyme (red, 144,934 data points) followed by membrane receptor (green, 113,793 data points), and ion channel (turquois, 16,023 data points). (B) for the L2 targets GPCRs are most dominant (green 104,668 data points), followed by proteases (red, 34,036 data points), and kinases (blue, 31,525 data points).*


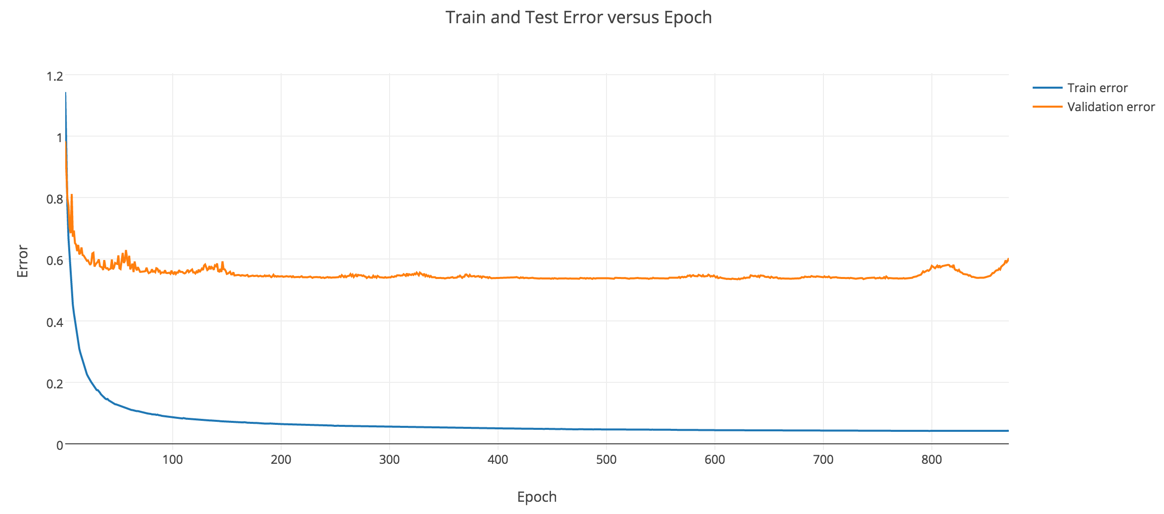


***Figure S8****: Representative plot for the training of the DNN. Here we have shown the training plot for the multi-task QSAR model using architecture 3 (8000, 4000, 2000 hidden nodes), trained on 4096 bits fingerprints with extra physicochemical properties. Shown is the training error (decreasing with epochs) and the validation error (weights are not updated). It can be seen that when the validation error increases (overtraining) the training stops.*

**Table S1: Results on the random split**

| Model | Global | | | Per Target | | |
| --- | --- | --- | --- | --- | --- | --- |
|  | BEDROC | ROC | MCC | BEDROC | ROC | MCC |
| PP NB MC 10 uM (NB 10 uM) | 0.717 | 0.644 | 0.200 | 0.612 | 0.632 | 0.180 |
| PP NB MC | 0.824 | 0.805 | 0.450 | 0.820 | 0.790 | 0.430 |
| PP RF QSAR | 0.930 | 0.863 | 0.580 | 0.812 | 0.799 | 0.450 |
| PP RF MC | 0.640 | 0.724 | 0.210 | 0.710 | 0.762 | 0.260 |
| PP RF PCM | 0.874 | 0.776 | 0.460 | 0.775 | 0.748 | 0.380 |
| PP SVM QSAR | 0.922 | 0.868 | 0.640 | 0.814 | 0.792 | 0.498 |
| PP LR QSAR | 0.686 | 0.636 | 0.319 | 0.540 | 0.558 | 0.129 |
| PY NB QSAR (NB) | 0.873 | 0.679 | 0.380 | 0.713 | 0.678 | 0.440 |
| PY RF QSAR (RF) | 0.795 | 0.868 | 0.630 | 0.850 | 0.825 | 0.490 |
| PY RF MC | 0.540 | 0.502 | 0.010 | 0.498 | 0.511 | 0.000 |
| PY RF PCM (RF_PCM) | 0.913 | 0.845 | 0.670 | 0.752 | 0.776 | 0.530 |
| PY DNN QSAR (DNN) | 0.956 | 0.879 | 0.600 | 0.861 | 0.813 | 0.460 |
| PY DNN MC (DNN_MC) | 0.962 | 0.890 | 0.630 | 0.871 | 0.831 | 0.500 |
| PY DNN PCM (DNN_PCM) | 0.962 | 0.894 | 0.610 | 0.894 | 0.840 | 0.480 |
| PY LR QSAR (LR) | 0.938 | 0.858 | 0.572 | 0.823 | 0.799 | 0.451 |
| PY SVM QSAR (SVM) | 0.927 | 0.858 | 0.572 | 0.826 | 0.790 | 0.436 |

*Shown is the mean Matthews correlation coefficient (MCC), BEDROC( α=20), and ROC when calculated per target, and these metrics when calculated for the merged predictions. Names in brackets indicate the name used throughout the main text.*

**Table S2: Results for the temporal split validation**

| Model | Global | | | Per Target | | |
| --- | --- | --- | --- | --- | --- | --- |
|  | BEDROC | ROC | MCC | BEDROC | ROC | MCC |
| PP NB MC 10 uM (NB 10 uM) | 0.654 | 0.588 | 0.000 | 0.550 | 0.566 | 0.000 |
| PP NB MC | 0.648 | 0.627 | 0.170 | 0.588 | 0.579 | 0.130 |
| PP RF QSAR | 0.490 | 0.643 | 0.290 | 0.571 | 0.569 | 0.140 |
| PP RF MC | 0.691 | 0.638 | 0.030 | 0.757 | 0.682 | 0.040 |
| PP RF PCM | 0.603 | 0.631 | 0.240 | 0.527 | 0.556 | 0.160 |
| PP SVM QSAR | 0.726 | 0.695 | 0.367 | 0.645 | 0.612 | 0.218 |
| PP LR QSAR | 0.536 | 0.548 | 0.145 | 0.518 | 0.509 | 0.020 |
| PY NB QSAR (NB) | 0.660 | 0.540 | 0.110 | 0.548 | 0.526 | 0.100 |
| PY RF QSAR (RF) | 0.505 | 0.643 | 0.260 | 0.570 | 0.569 | 0.090 |
| PY RF MC | 0.490 | 0.500 | -0.010 | 0.490 | 0.510 | -0.010 |
| PY RF PCM (PCM) | 0.603 | 0.631 | 0.210 | 0.526 | 0.556 | 0.060 |
| PY DNN QSAR (DNN) | 0.793 | 0.718 | 0.300 | 0.665 | 0.622 | 0.140 |
| PY DNN MC (DNN_MC) | 0.823 | 0.724 | 0.330 | 0.707 | 0.654 | 0.190 |
| PY DNN PCM (DNN_PCM) | 0.848 | 0.733 | 0.330 | 0.706 | 0.658 | 0.200 |
| PY LR QSAR (LR) | 0.737 | 0.670 | 0.256 | 0.616 | 0.591 | 0.108 |
| PY SVM QSAR (SVM) | 0.731 | 0.695 | 0.293 | 0.642 | 0.608 | 0.143 |

*Shown is the mean Matthews correlation coefficient (MCC), BEDROC( α=20), and ROC when calculated per target, and these, metrics when calculated for the merged predictions. Names in brackets indicate the name used throughout the main text.*

**Table S3: Results for the training time in hours.**

| Model | Training time |
| --- | --- |
| PP NB MC-QSAR 10 uM (NB 10 uM) | 0.11 |
| PP NB MC-QSAR | 31.2 |
| PP RF QSAR | 2.3 |
| PP RF MC-QSAR | 141.6 |
| PP RF PCM | 14 |
| PP SVM QSAR | 5.93 |
| PP LR QSAR | 5.27 |
| PY NB QSAR (NB) | 0.3 |
| PY RF QSAR (RF) | 4.2 |
| PY RF MC-QSAR | 6.1 |
| PY RF PCM (RF_PCM) | 2.35 |
| PY DNN QSAR (DNN) | 5.4 |
| PY DNN MC-QSAR (DNN_MC) | 9.6 |
| PY DNN PCM (DNN_PCM) | 8.9 |
| PY LR QSAR (LR) | 0.02 |
| PY SVM QSAR (SVM) | 0.05 |

*Names in brackets indicate the name used throughout the main text.*

**Table S4: overview of p values obtained with Students T test.**

| **Method (Student T)** | **NB 10 uM** | **NB** | **RF** | **RF_PCM** | **SVM** | **LR** | **DNN** | **DNN_MC** | **DNN_PCM** |
| --- | --- | --- | --- | --- | --- | --- | --- | --- | --- |
| NB 10 uM | 1.00 | 0.06 | 0.03 | 0.03 | 0.01 | 0.01 | 0.01 | 0.00 | 0.00 |
| NB |  | 1.00 | 0.40 | 0.33 | 0.00 | 0.00 | 0.00 | 0.00 | 0.00 |
| RF |  |  | 1.00 | 0.91 | 0.24 | 0.32 | 0.12 | 0.06 | 0.05 |
| RF_PCM |  |  |  | 1.00 | 0.29 | 0.38 | 0.14 | 0.07 | 0.06 |
| SVM |  |  |  |  | 1.00 | 0.36 | 0.09 | 0.01 | 0.04 |
| LR |  |  |  |  |  | 1.00 | 0.03 | 0.01 | 0.02 |
| DNN |  |  |  |  |  |  | 1.00 | 0.11 | 0.16 |
| DNN_MC |  |  |  |  |  |  |  | 1.00 | 0.86 |
| DNN_PCM |  |  |  |  |  |  |  |  | 1.00 |

*Values are based on the MCC and BEDROC z-scores for both temporal and random split experiments. Color thresholds are 0.05 (green), 0.07 (white), and 0.3 (red). Values in between are graded in between the colors (e.g. light green for 0.09).*

**Table S5: overview of p values obtained with Fisher F test.**

| **Method (Fisher F)** | **NB 10 uM** | **NB** | **RF** | **RF_PCM** | **SVM** | **LR** | **DNN** | **DNN_MC** | **DNN_PCM** |
| --- | --- | --- | --- | --- | --- | --- | --- | --- | --- |
| NB 10 uM | 1.00 | 0.00 | 0.95 | 0.95 | 0.03 | 0.01 | 0.06 | 0.10 | 0.26 |
| NB |  | 1.00 | 0.00 | 0.00 | 0.25 | 0.49 | 0.15 | 0.10 | 0.03 |
| RF |  |  | 1.00 | 0.99 | 0.03 | 0.01 | 0.05 | 0.09 | 0.23 |
| RF_PCM |  |  |  | 1.00 | 0.03 | 0.01 | 0.06 | 0.09 | 0.24 |
| SVM |  |  |  |  | 1.00 | 0.63 | 0.74 | 0.54 | 0.24 |
| LR |  |  |  |  |  | 1.00 | 0.42 | 0.29 | 0.11 |
| DNN |  |  |  |  |  |  | 1.00 | 0.78 | 0.38 |
| DNN_MC |  |  |  |  |  |  |  | 1.00 | 0.54 |
| DNN_PCM |  |  |  |  |  |  |  |  | 1.00 |

*Values are based on the MCC and BEDROC z-scores for both temporal and random split experiments. Color thresholds are 0.05 (green), 0.07 (white), and 0.3 (red). Values in between are graded in between the colors (e.g. light green for 0.06).*

**Table S6: overview of p values obtained with Wilcoxon signed rank test.**

| **Method (Wilcoxon)** | **NB 10 uM** | **NB** | **RF** | **RF_PCM** | **SVM** | **LR** | **DNN** | **DNN_MC** | **DNN_PCM** |
| --- | --- | --- | --- | --- | --- | --- | --- | --- | --- |
| NB 10 uM | 1.00 | 0.06 | 0.06 | 0.06 | 0.03 | 0.03 | 0.03 | 0.03 | 0.03 |
| NB |  | 1.00 | 0.34 | 0.34 | 0.03 | 0.03 | 0.03 | 0.03 | 0.03 |
| RF |  |  | 1.00 | 1.00 | 0.34 | 0.34 | 0.11 | 0.03 | 0.06 |
| RF_PCM |  |  |  | 1.00 | 0.34 | 0.34 | 0.34 | 0.11 | 0.06 |
| SVM |  |  |  |  | 1.00 | 0.49 | 0.11 | 0.03 | 0.06 |
| LR |  |  |  |  |  | 1.00 | 0.06 | 0.03 | 0.03 |
| DNN |  |  |  |  |  |  | 1.00 | 0.11 | 0.20 |
| DNN_MC |  |  |  |  |  |  |  | 1.00 | 0.89 |
| DNN_PCM |  |  |  |  |  |  |  |  | 1.00 |

*Values are based on the MCC and BEDROC z-scores for both temporal and random split experiments. Color thresholds are 0.05 (green), 0.07 (white), and 0.3 (red). Values in between are graded in between the colors (e.g. light green for 0.06).*

**Table S7: overview of p values obtained with Kolgomorov – Smirnov test.**

| **Method (Kolgomorov)** | **NB 10 uM** | **NB** | **RF** | **RF_PCM** | **SVM** | **LR** | **DNN** | **DNN_MC** | **DNN_PCM** |
| --- | --- | --- | --- | --- | --- | --- | --- | --- | --- |
| NB 10 uM | 1.00 | 0.23 | 0.23 | 0.23 | 0.03 | 0.03 | 0.03 | 0.03 | 0.03 |
| NB |  | 1.00 | 0.23 | 0.23 | 0.03 | 0.03 | 0.03 | 0.03 | 0.03 |
| RF |  |  | 1.00 | 1.00 | 0.23 | 0.23 | 0.23 | 0.03 | 0.23 |
| RF_PCM |  |  |  | 1.00 | 0.23 | 0.23 | 0.23 | 0.23 | 0.23 |
| SVM |  |  |  |  | 1.00 | 0.77 | 0.23 | 0.03 | 0.23 |
| LR |  |  |  |  |  | 1.00 | 0.23 | 0.03 | 0.03 |
| DNN |  |  |  |  |  |  | 1.00 | 0.23 | 0.23 |
| DNN_MC |  |  |  |  |  |  |  | 1.00 | 1.00 |
| DNN_PCM |  |  |  |  |  |  |  |  | 1.00 |

*Values are based on the MCC and BEDROC z-scores for both temporal and random split experiments. Color thresholds are 0.05 (green), 0.07 (white), and 0.3 (red). Values in between are graded in between the colors (e.g. light red for 0.23).*

**Table S8: Impact of including inactive data points on the Matthew’s Correlation Coefficient (MCC).**

| Test Set | MCC (Actives) | MCC (Inactives & Actives) |
| --- | --- | --- |
| 90% | 0.22 | 0.31 |
| 70% | 0.25 | 0.42 |
| 50% | 0.27 | 0.45 |
| 30% | 0.27 | 0.47 |
| 10% | 0.29 | 0.54 |

*The second column method used only z-scores of active targets, while the third column method subtracted the z-scores of inactive targets from the active targets. This was done for different test set split (10%, 30%, 50%, 70%, 90% training data respectively).*

**References**

1. Landrum G. RDKit: Cheminformatics and Machine Learning Software 2013.

2. Rogers D, Hahn M. Extended-connectivity fingerprints. J Chem Inf Model. 2010;50(5):742-54.

3. Landrum G. Fingerprints in the RDKit.

4. Mugumbate G, Abrahams KA, Cox JAG, Papadatos G, van Westen G, LeLièvre J, et al. Mycobacterial Dihydrofolate Reductase Inhibitors Identified Using Chemogenomic Methods and In Vitro Validation. PLoS One. 2015;10:e0121492.

5. Martínez-Jiménez F, Papadatos G, Yang L, Wallace IM, Kumar V, Pieper U, et al. Target Prediction for an Open Access Set of Compounds Active against Mycobacterium tuberculosis. PLoS Comput Biol. 2013;9:e1003253.

6. van Westen GJ, Swier RF, Cortes-Ciriano I, Wegner JK, Overington JP, Ijzerman AP, et al. Benchmarking of protein descriptor sets in proteochemometric modeling (part 2): modeling performance of 13 amino acid descriptor sets. J Cheminform. 2013;5(1):42.

7. Van Westen GJ, Wegner JK, Geluykens P, Kwanten L, Vereycken I, Peeters A, et al. Which compound to select in lead optimization? Prospectively validated proteochemometric models guide preclinical development. PloS one. 2011;6(11):e27518.
